# Investigation of Yield Surfaces Evolution for Polycrystalline Aluminum after Pre-Cyclic Loading by Experiment and Crystal Plasticity Simulation

**DOI:** 10.3390/ma13143069

**Published:** 2020-07-09

**Authors:** Damin Lu, Keshi Zhang, Guijuan Hu, Yongting Lan, Yanjun Chang

**Affiliations:** 1 Key Lab of Disaster Prevent and Structural Safety, Guangxi Key Lab Disaster Prevent and Engineering Safety, College of Civil Engineering and Architecture, Guangxi University, Nanning 530004, China; ludamin@st.gxu.edu.cn (D.L.); zhangks@gxu.edu.cn (K.Z.); changyj@gxu.edu.cn (Y.C.); 2 School of Landscape Architecture, Zhejiang A & F University, Hangzhou 311300, China; 3 School of Vocational and Technical Education, Guangxi University of Science and Technology, Liuzhou 545006, China; 100000877@gxust.edu.cn

**Keywords:** yield surface, crystal plasticity, anisotropic hardening, polycrystalline aluminum

## Abstract

This study aims at introducing the back stress of anisotropic strain-hardening into the crystal plasticity theory and demonstrating the rationality of this crystal plasticity model to describe the evolution of the subsequent yield surface of polycrystalline aluminum at the mesoscopic scale under complex pre-cyclic loading paths. By using two different scale finite element models, namely a global finite element model (GFEM) as the same size of the thin-walled tube specimen used in the experiments and a 3D cubic polycrystalline aggregate representative volume element (RVE) model, the evolution of the subsequent yield surface for different unloading cases after 30 pre-cycles is further performed by experiments and numerical simulations within a crystal plasticity finite element (CPFE) frame. Results show that the size and shape of the subsequent yield surfaces are extremely sensitive to the chosen offset strain and the pre-cyclic loading direction, which present pronounced anisotropic hardening through a translation and a distortion of the yield surface characterized by the obvious “sharp corner” in the pre-deformation direction and “flat” in the reverse direction by the definition of small offset strain, while the subsequent yield surface exhibits isotropic hardening reflected by the von Mises circle to be distorted into an ellipse by the definition of large offset strain. In addition, the heterogeneous properties of equivalent plastic strain increment are further discussed under different offset strain conditions. Modeling results from this study show that the heterogeneity of plastic deformation decreases as a law of fraction exponential function with the increasing offset strain. The above analysis indicates that anisotropic hardening of the yield surface is correlated with heterogeneous deformation caused by crystal microstructure and crystal slip. The crystal plasticity model based on the above microscopic mechanism can accurately capture the directional hardening features of the yield surface.

## 1. Introduction

Polycrystalline materials often exhibit more complicated plastic anisotropy during the forming and manufacturing processes of metal components [1,2]. The initial anisotropy induced by plastic deformations gives rise to the complex distorted evolution and translation of subsequent yield surfaces under complex pre-loading paths, which include cyclic loading histories. Under the monotonous proportional and non-proportional pre-loading paths, a series of studies has already demonstrated that the subsequent yield surface will change size, location, and shape [3,4,5,6,7,8], which are derived from the isotropic, kinematic, and distortional hardening mechanisms in plastic constitutive models [9]. Under the pre-cyclic loading paths, a few studies of the yield surfaces were performed [10,11] and the results showed that cyclic hardening, softening, ratcheting, Bauschinger effect, and cyclic strain amplitude resulted in a complex evolution of subsequent yield surfaces. For these complex subsequent yielding phenomena of materials, the study of yield surface evolution is the key to establish a reasonable constitutive model to describe metal plastic behavior accurately under complex pre-loading paths [10,12].

In general, the shape of the yield surface for most metals is sensitive to the yield definition. Numerous experimental results have shown the distorted evolution of the subsequent yield surfaces characterized by a triangle vertex in the loading direction but perfectly flattening in the reverse loading direction by yield definition of small offset strain [3,5,6,7,13], while the subsequent yield surface based on large offset strain value is closer to von-Mises surface [14,15]. For instance, using the same experimental materials as stainless steel 304, the shape of subsequent yield surfaces becomes an ellipse with the definition of yield as 50 με offset strain [16,17], but exhibits directional distortion with the definition of yield as 5 με offset strain [3]. The previous experimental studies about the yield surface were performed by the smaller proof strain in the range of 5–50 με using a single specimen method, which all yield points on a subsequent yield surface were determined by a thin-walled tube specimen. Because the accumulated plastic strain caused by the previous yield points probed and the accuracy of measuring instrument is negligible, some yield points on the subsequent yield surface by a single specimen methods may be have large deviation, but numerical simulations and multi-specimen test method can almost eliminate the effect of these factors using this inaccurate measurement methods. The experimental studies of the yield surfaces using multiple sample methods are rarely reported with various offset strain ranging from 20 με to 1000 με.

Based on experimental observations, an early documented attempt to describe the macroscopic anisotropy yielding of metals introduced the appropriate kinematic and distortional hardening rules, based on the classical isotropic hardening, into plasticity theories [18,19,20,21,22,23]. For instance, a non-quadratic yield surface had been adopted for modeling the anisotropic responses of aluminium alloys [24,25]. Some researchers introduced a distortional stress tensor into the von Mises yield criterion, and then the yield surface described as an “egg shape” [21]. In this study, the hardening rules were derived from thermodynamics for both the back-stress and the fourth order tensor. These macroscopic continuum models make no attempt to incorporate the microscopic behavior of the material, and the yield functions are defined by the stress field and a set of internal variables by the choice of distortion parameters. Most of the above models predict the von Mises circle to be distorted into an ellipse or a “round nose” rather than to become high curvature of yield surface distortion as experimental observations. The evolution of the yield surface depends markedly on the plastic slip mechanisms and the crystallographic texture for polycrystal material [25]. The crystal plasticity model can be employed to accurately describe the anisotropic evolution of the subsequent yield surfaces [26,27].

In this work, the evolution of the subsequent yield surfaces for different unloading cases with various yield definitions after different pre-cyclic loading is studied in detail by both experiments and crystal plasticity simulations using multiple identical specimens. The work is highlighted within a crystal plasticity finite element (CPFE) scheme using two different scale models: a thin-walled circular tube finite element model of the same size as the test specimen and a representative 3D cubic polycrystalline aggregate representative volume element RVE model. Furthermore, by comparing the experimental data with the numerical simulations, the ability of the crystal plasticity model considering a back stress for anisotropic strain-hardening to describe the anisotropic evolution of the subsequent yield surfaces under the loading path change and different yield definition is validated.

## 2. Material and Experimental Procedure

### 2.1. Experimental Material and Specimen

**Figure 1:**
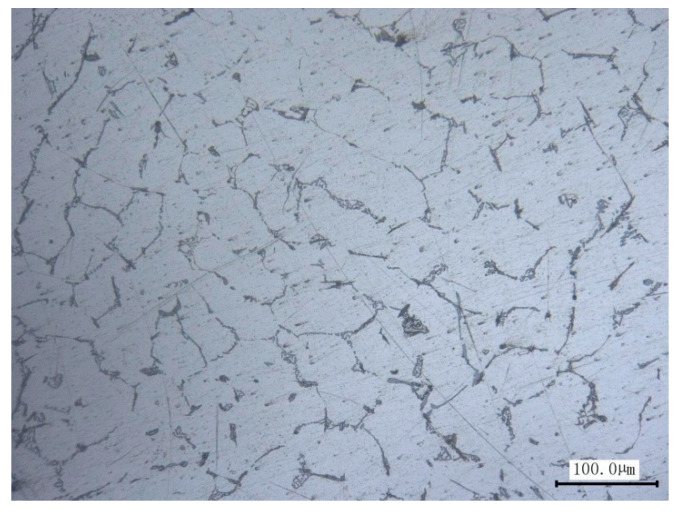
Metallographic image of the polycrystal aluminum.

**Figure 2:**
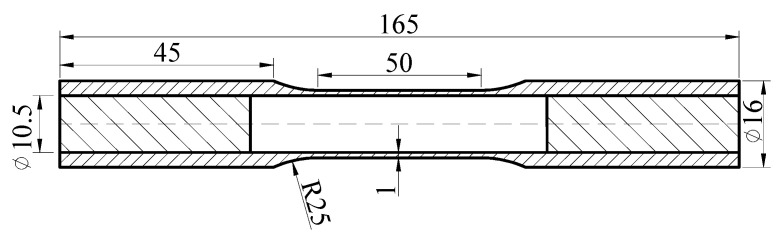
Specimen geometry of pure aluminum (unit: mm).

The material used in this study is the high-purity (99.89%) polycrystalline aluminum, and the metallographic photo shows an equiaxed grain structure and average grain size is proximately 80 μm in Figure 1. Its chemical composition and the basic mechanical properties from uniaxial tension test and pure torsion test are given in Table 1. These experiments are carried out by thin-walled tube specimens made of aluminum alloy materials subjected to combined axial-torsional loading, the geometry of the specimens is shown in Figure 2. The steel inserts are embedded in both ends of the specimens to ensure reliable clamping and resist the severe deformation.

### 2.2. Experimental Procedure

**Figure 3:**
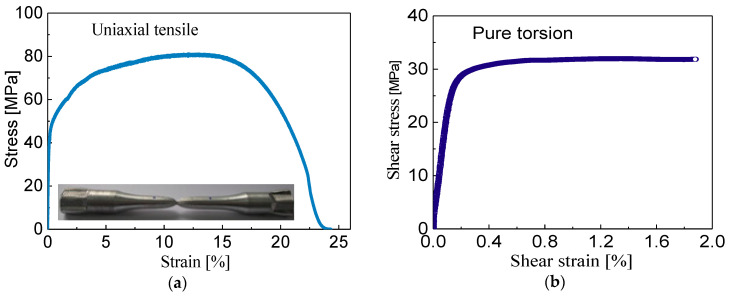
Stress-strain curves of polycrystalline aluminum by test: (**a**) the stress-strain response in the uniaxial tension, (**b**) the stress-strain response in the pure torsion.

The experiments are conducted by an MTS809 tension-torsional servo-controlled hydraulic testing machine (MTS Systems Corporation, Eden Prairie, MN, USA) at room temperature. An extensometer with a 25-mm gauge length is calibrated to measure the axial and torsion strain of the gauge section of specimens. The stress-strain curves of σ(ε) in uniaxial tension and τ(γ) in pure torsion are displayed in Figure 3. The stress–strain behaviour under uniaxial tensile loading exhibits the pronounced strain hardening effect in which a high strain-hardening rate is observable with the increase of axial strain. Unlike the axial component of flow stress, the stress–strain curve obtained from the pure torsion experiment shows the lower strain hardening behavior.

**Figure 4:**
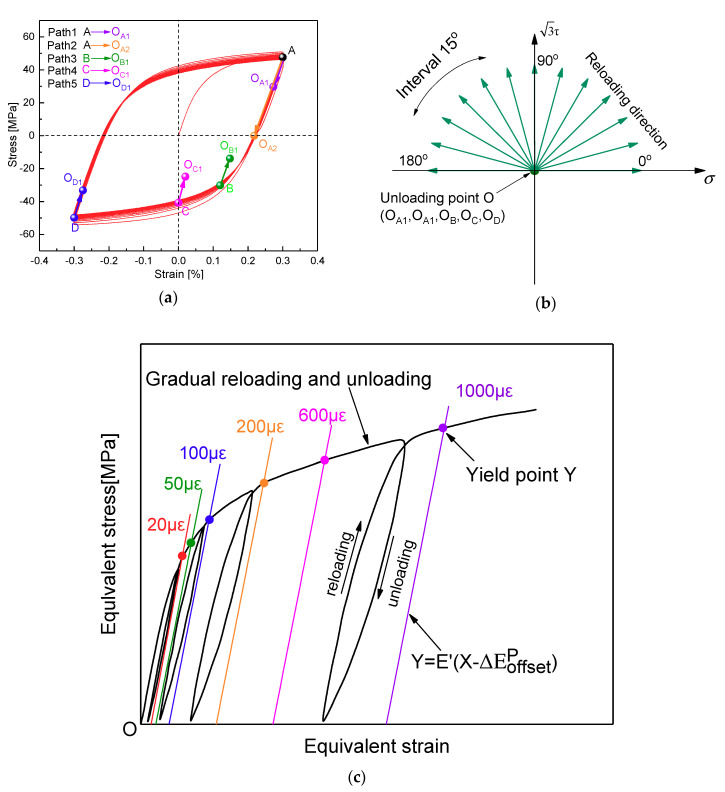
Preloading, unloading and reloading paths for probing the subsequent yield surface by experiments and numerical simulations: (**a**) different unloading end positions after pre-cyclic tension-compression loading, (**b**) reloading direction under combined tension-torsion proportional load, (**c**) Schematic diagram of yield point definition for yield surface.

The subsequent yield behaviors of polycrystal aluminum with the multiple-specimen method are studied at five different unloading cases (A → O_A1_, A → O_A2_, B → O_B_, C → O_C_, D → O_D_) under pre-cyclic loading paths by experiment and the crystal plasticity finite element scheme, and the unloading points O_A1_, O_A2_, O_B_, O_C_, or O_D_ were defined as the center of the yield surface, respectively. The determined processes of the subsequent yield surfaces are mainly summarized as the following three steps. First (pre-cyclic loading process), each specimen is cyclically loaded to a steady stress state after 30 symmetrical tensile-compressive cycles at the 0.3% cyclic strain amplitude with the strain-controlled mode in Figure 4a. Then (unloading process), for each unloading case, the specimen is unloaded from different locations (A, B, C, or D) on a saturation hysteretic loop to the unloading end points (O_A1_, O_A2_, O_B_, O_C_ or O_D_) under the stress-controlled mode respectively, as shown in Figure 4a (A → O_A1_, A → O_A2_, B → O_B_, C → O_C_, D → O_D_). It is worth noting specially that all unloading locations should be limited within the elastic range, or the lower yield strength caused by reverse loading will change the size of yield surface. Finally (reloading process), the specimen is reloaded along a specific reloading direction, which is represented by a radial ling in Figure 4b, to determine the yield points of subsequent yield surfaces with different target offset strain by combined tension-torsion proportional loading paths ranging from 0° to 180° at intervals of 15°.

The definition of yield points adopted here is the unloading stiffness method, considering the unloading stiffness decreases with the increase of offset strain [5,6,7], The unloading stiffness E’ corresponding to each offset strain is determined by the method of gradual reloading and unloading, as shown in Figure 4c. First, the specimen is reloaded from the unloading point to the stress state approach to the target offset strain $\Delta E_{\mathrm{offset}}^{p}$, then the specimen is unloaded back to the unloading point, and the unloading stiffness E′ is determined by fitting the linear portion of the unloading curve. By drawing a straight line $Y=E^{\prime }(X-\Delta E_{\mathrm{offset}}^{p})$, which parallels the unloading stiffness E′, the intersection of the straight line and the equivalent stress–strain curve is defined as the subsequent yield point represented by Y. By repeating this reloading–unloading process, the subsequent yield points corresponding to each given target strain 20 με, 50 με, 100 με, 200 με, 600 με, and 1000 με in each reloading direction can be determined completely.

## 3. Constitutive Relationships and Coupling Methodology

### 3.1. Constitutive Framework

A crystalline plastic model is used which considers the typical 12 slip systems for a single crystal of face centered cubic (FCC) structure materials. The total deformation gradient within the crystal plasticity framework can be multiplicatively decomposed as:

(1) $$F=F^{*}\cdot F^{p}$$

where $F^{*}$ is the elastic component arising from elastic stretching $U^{e}$ and rigid body rotation $R^{L}$ of the configuration according to the standard polar decomposition, which can be expressed as: $F^{*}=R^{L}\cdot U^{e}$. $F^{p}$ is the plastic component describing the dislocation glide along the specific slip planes [28].

For a material point in a crystal grain, the Euler’s velocity gradient tensor L can then be defined as the sum of the skew-symmetric gradient tensor $L^{*}$ and the plastic gradient tensor $L^{p}$ [29,30,31]:

(2) $$L=\dot{F}\cdot F^{-1}=L^{*}+L^{p}=\dot{F}{}^{*}\cdot F^{{*}^{-1}}+F^{*}\cdot \dot{F}{}^{p}\cdot F^{p^{-1}}\cdot F^{{*}^{-1}}$$

The Euler’s velocity gradient tensor L can be further divided into a symmetric deformation velocity tensor, $D=\mathrm{sym}(L)=\frac{1}{2}\left(L+L^{T}\right)$, and the anti-symmetric rotation velocity tensor, $W=\mathrm{asym}(L)=\frac{1}{2}\left(L-L^{T}\right)$, where $L^{T}$ denotes a ‘transpose’ matrix of the velocity gradient tensor. Further, we write:

(3) L=D+W

Subsequently, the deformation tensor and rotation tensor, respectively, can be decomposed into a lattice contribution elastic part and a plastic part [32]:

(4) $$D=D^{*}+D^{p}=\mathrm{sym}\left(L^{*}\right)+\mathrm{sym}\left(L^{p}\right)$$

(5) $$W=W^{*}+W^{p}=\mathrm{asym}\left(L^{*}\right)+\mathrm{asym}\left(L^{p}\right).$$

where $D^{*}$ and $D^{p}$ are, respectively, the elastic part and the plastic part of stretching tensor. $W^{p}$ is the plastic spin tensor generated by crystal slip and $W^{*}$ is the lattice spin tensor, which can be defined as follows [33]:

(6) $$W^{*}=R\cdot \dot{R}{}^{T}$$

where R is the rigid body rotation of crystal lattice, $\dot{R}{}^{T}$ is elastic distortion.

The plastic velocity gradient in the intermediate (relaxed) configuration, $L^{p}$, is defined as the sum of the reference shear strain rate $\dot{\gamma }{}^{\left(\alpha \right)}$ on all slip systems [34]:

(7) $$L^{p}=\dot{F}{}^{p}\cdot F^{p^{-1}}=\sum_{\alpha =1}^{n}(m^{\left(\alpha \right)}n^{\left(\alpha \right)})\dot{\gamma }{}^{\left(\alpha \right)}=\sum_{\alpha =1}^{n}s^{\left(\alpha \right)}\dot{\gamma }{}^{\left(\alpha \right)}$$

where the term ($s^{\left(\alpha \right)}=m^{\left(\alpha \right)}n^{\left(\alpha \right)}$) represents the Schmidt tensor on the *α*-th slip system that is defined by the dyadic product of orthogonal unit vectors $m^{\left(\alpha \right)}$ and $n^{\left(\alpha \right)}$ in the reference configuration, with $m^{\left(\alpha \right)}$ indicating the slip direction and $n^{\left(\alpha \right)}$ being the normal to the slip plane. $\dot{\gamma }{}^{\alpha }$ is a reference shear strain rate on the *α*-th slip system, n is the total number of slip systems.

When the deformation gradient $F^{*}$ is applied, the crystalline lattice rotates and these vectors ($m^{\left(\alpha \right)}$ and $n^{\left(\alpha \right)}$) in the reference configuration transform to $m^{(\alpha )*}$ and $n^{(\alpha )*}$ respectively, in the current deformed configuration, such that:

(8) $$m^{(\alpha )*}=F^{*}\cdot m^{\left(\alpha \right)}$$

(9) $$n^{(\alpha )*}=n^{\left(\alpha \right)}\cdot F^{{*}^{-1}}$$

where it is assumed that $m^{\left(\alpha \right)}$ and $n^{\left(\alpha \right)}$ are orthogonal to each other.

(10) $$m^{(\alpha )*}\cdot n^{(\alpha )*}=m^{\left(\alpha \right)}\cdot n^{\left(\alpha \right)}=0$$

The Schmid tensor on the *α*-th slip system can be written as the sum of asymmetric tensor $p^{\left(\alpha \right)}$ and an anti-symmetric tensor $\omega^{\left(\alpha \right)}$, such that:

(11) $$s^{\left(\alpha \right)}=p^{\left(\alpha \right)}+\omega^{\left(\alpha \right)}$$

where the symmetric part and anti-symmetric part of the Schmid tensor on the *α*-th slip system can be defined as:

(12) $$p^{\left(\alpha \right)}=\frac{1}{2}(I^{\left(\alpha \right)}+{\left(I^{\left(\alpha \right)}\right)}^{T})=\frac{1}{2}(m^{(\alpha )*}n^{(\alpha )*}+n^{(\alpha )*}m^{(\alpha )*})$$

(13) $$\omega^{\left(\alpha \right)}=\frac{1}{2}(I^{\left(\alpha \right)}-{\left(I^{\left(\alpha \right)}\right)}^{T})=\frac{1}{2}(m^{(\alpha )*}n^{(\alpha )*}-n^{(\alpha )*}m^{(\alpha )*})$$

The plastic deformation gradient can be expressed as:

(14) $$D^{p}=\frac{1}{2}\left(L^{p}+L^{\mathrm{pT}}\right)=\sum_{\alpha =1}^{n}p^{\left(\alpha \right)}\dot{\gamma }{}^{\left(\alpha \right)}$$

Similarly, the spin tensor, which represents the material rotation due to slip, can be expressed as:

(15) $$W^{p}=\frac{1}{2}\left(L^{p}-L^{\mathrm{pT}}\right)=\sum_{\alpha =1}^{n}\omega^{\left(\alpha \right)}\dot{\gamma }{}^{\left(\alpha \right)}$$

Further, the elastic stretching tensor and the lattice spin tensor can be written as:

(16) $$D^{*}=\frac{1}{2}\left(L^{*}+L^{*T}\right)=D-\sum_{\alpha =1}^{n}p^{\left(\alpha \right)}\dot{\gamma }{}^{\left(\alpha \right)}$$

(17) $$W^{*}=\frac{1}{2}\left(L^{*}-L^{*T}\right)=W-\sum_{\alpha =1}^{n}\omega^{\left(\alpha \right)}\dot{\gamma }{}^{\left(\alpha \right)}$$

### 3.2. Hardening Rules

In this work, considering the predominant cyclic strain preloading paths in the subsequent yield problem, while the effects of back-stress are essential to be considered for the case of cyclic loading. Accordingly, the conventional crystalline plastic theory has been developed to capture the cyclic plastic response of the alloy crystals [12]. According to the viscous regularization proposed by Hutchinson [35], an additional back-stress $x^{\left(\alpha \right)}$ is here introduced into the viscoplastic constitutive model. The slip rate for a given slip system is given by:

(18) $${\dot{\gamma }}^{\left(\alpha \right)}={\dot{\gamma }}_{0}\mathrm{sgn}(\tau^{\left(\alpha \right)}-x^{\left(\alpha \right)}){\left|\frac{\tau^{\left(\alpha \right)}-x^{\left(\alpha \right)}}{g^{\left(\alpha \right)}}\right|}^{k}$$

where ${\dot{\gamma }}_{0}$ is a reference shear strain rate on the *α*-th slip system, k is the power law exponent representing the rate sensitivity, $\tau^{\left(\alpha \right)}$ is the resolved shear stress on the *α*-th slip system of the single crystal, $g^{\left(\alpha \right)}$ is the critical shear stress on the activated slip system *α* to govern the isotropic hardening of the crystal, and the back-stress $x^{\left(\alpha \right)}$ is introduced here to characterize the nonlinear directional hardening of the crystal on the *α*-th slip system.

The evolution of the critical shear stress $g^{\left(\alpha \right)}$ for a given activated slip system *α* is described as:

(19) $${\dot{g}}^{\left(\alpha \right)}(\gamma )=\sum_{\beta =1}^{n}h_{\alpha \beta }(\gamma )\left|{\dot{\gamma }}^{\left(\beta \right)}\right|;\gamma =\int \sum_{\beta }^{n}\left|d\gamma^{\left(\beta \right)}\right|$$

where $h_{\alpha \beta }(\gamma )$ is the slip-plane hardening modulus which governs the interaction between various slip systems, $\gamma^{\left(\beta \right)}$ is the slip rate on the system *β*, *n* is the number of crystal slip systems, and γ is the accumulated shear strains on the all active slip systems. Moreover, Hutchinson suggested [36]:

(20) $$h_{\alpha \beta }(\gamma )=h(\gamma )[q+(1-q)\delta_{\alpha \beta }]$$

where *q* is a constant, whose value can be chosen in the range from 0 to 1, and Chang and Asaro suggested [37]:

(21) $$h(\gamma )=h_{0}\sec h^{2}\left(\frac{h_{0}\gamma }{\tau_{s}-\tau_{0}}\right)$$

where $h_{0}$ is the initial hardening rate, $\tau_{0}$ is the critical resolved shear stress and $\tau_{s}$ is the saturation shear stress, which can be preliminarily determined according to elastic range of the experimental hysteresis loop.

### 3.3. Back Stress Evolution

According to Walker [38] and the Chaboche model [39], the evolution of back-stress ${\dot{x}}^{\left(\alpha \right)}$ is expressed as [10,32].

(22) $${\dot{x}}^{\left(\alpha \right)}=a{\dot{\gamma }}^{\left(\alpha \right)}-c[1-e_{1}(1-\exp(-e_{2}\gamma ))]x^{\left(\alpha \right)}\left|{\dot{\gamma }}^{\left(\alpha \right)}\right|-dx^{\left(\alpha \right)}$$

where a is the initial hardening modulus of the slip system, c and d are the nonlinear hardening parameters, respectively, $e_{1}$ and $e_{2}$ are used to describe the law of the cyclic hardening saturation. The above material parameters which describe the cyclic plastic behavior are determined by the trial-and-error method combined with cyclic tests.

The above constitutive relation has been implemented into the user material subroutine UMAT for the ABAQUS (Dassault Systems, Paris, France)/Standard module [10,32].

## 4. Modeling

### 4.1. Finite Element Models at Different Scale

**Figure 5:**
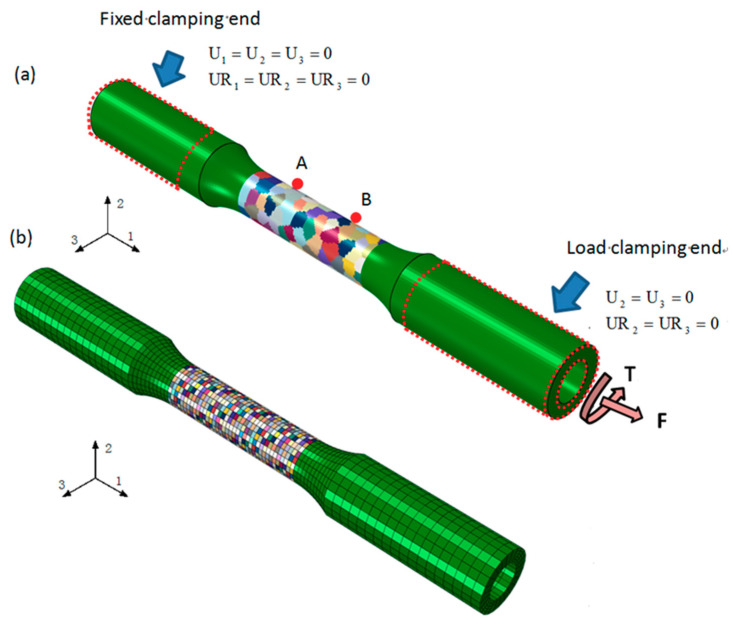
The FEM (Finite Element Model) of thin-walled tube specimen: (**a**) a grain contain multiple elements; (**b**) each element standards for a grain.

To treat the deformation behavior of micro-inhomogeneity at the grain scale, the finite element model (FEM) of polycrystal aluminum within crystal plasticity framework is built using a global finite element model (GFEM) as the same size of the thin-walled tube specimen used in the experiments. Two different representative models of the microstructure, which the GFEM consists of the same 76,000 C3D8 elements and different crystalline grains in the center gauge region, are used, as shown in Figure 5. The first one (GFEM) is shown in Figure 5a, where each grain as polycrystalline aggregate is composed of many elements with random shape, size, and random crystal orientations [40]. It is further demonstrated that this size is accurate enough of the statistical representation of the actual microstructure for obvious different number of grains in the center gauge region by sequential serial sectioning. Another one (GFEM) is built, as shown in Figure 5b, in which each cubic element standards for a grain [27,40]. Thus, the FEM model is divided into a great deal of grains resulted in a poor description of the grain shape.

For a GFEM model, the macroscopic axial strain and torsion strain in each increment are calculated by outputting the axial displacement U_1_ and torsion displacement U_R1_ at the reference point A and B simulating an axial-torsional extensometer of 25 mm fixed on the specimen’s gauge section in the test. The model is constrained and loaded by coupling the nodes in fixed clamping end and load clamping end with their respective reference point, and the macroscopic loading conditions are introduced in the model by applying a axial load F and a torsion load T in the reference point of load clamping end in order to achieve the axial and torsional deformation history, as shown in Figure 5a.

**Figure 6:**
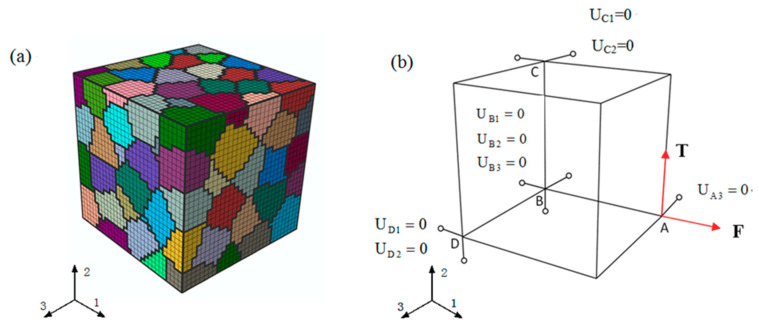
The representative volume element (RVE) model containing 125 grains and the loading boundary condition: (**a**) RVE model; (**b**) loading boundary condition.

In this research, the finite element model (FEM) for polycrystal aluminum is also built using a 3D cubic polycrystal RVE (cube of side 1 mm) with polycrystalline aggregate as a material point to simulate the gauge section of a thin-walled tube by the crystal plasticity model, as shown in Figure 6. The depicted RVE contains 27,000 elements and is divided into 125 equiaxed grains. The grains in the above FEM are given attributes with random morphology, size, and random crystal orientations, and the RVE is treated as a macroscopic isotropic body. The boundary conditions of loading and displacement are exerted to the RVE in three faces along the direction of $x_{k}\left(k=1,2,3\right)$, respectively, shown in Figure 6b. In addition to using the equation constraints of ABAQUS software to constrain all nodes on the surface of the representative element body to meet the periodic boundary conditions [39,40], four nodes A, B, C, and D of the RVE are selected in the representative element body as reference points and the corresponding macroscopic constraints are applied to the element body in Figure 6b. In order to reflect the combined axial-torsional loading conditions as the experiments for the RVE model, macro axial force F and macro lateral torque T are applied to the reference point A on the loading surface relative to the fixed surface.

### 4.2. Parameters Calibration of Crystalline Plasticity Models

**Figure 7:**
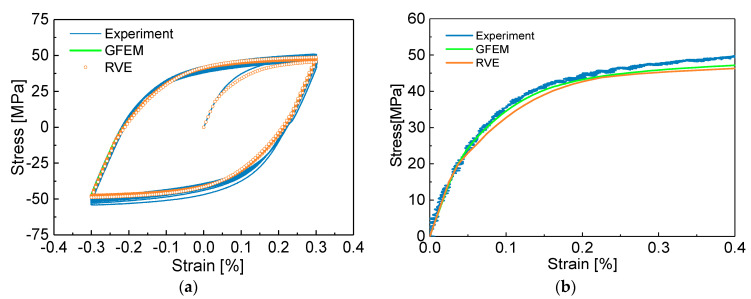
Stress-strain curve of tension-compression cyclic loading and monotonic tension curve by test and crystal plasticity simulation with the global finite element model (GFEM) containing 36,000 grains as the same size of the thin-walled tube specimen used in the experiments and the RVE containing 125 grains: (**a**) cyclic stress-strain curve; (**b**) monotonic tension curve.

Material parameters for crystal plasticity are calibrated by a GFEM containing 36,000 elements and 36,000 crystalline grains in the center gauge region and the RVE consisting of 27,000 elements and 125 equiaxed grains by crystalline plasticity, respectively. Figure 7a shows that the cyclic stress-strain curve tends to saturate after 30 cycles during the tension-compression loading at 0.3% strain amplitude by experiment, in other words, the polycrystalline aluminum exhibits stable cyclic hardening behavior. Therefore, the constitutive parameters of crystalline plasticity are calibrated by fitting the predicted stable hysteresis curve with the experimental data in Figure 7a. The constitutive parameters are further adjusted from the monotonic uniaxial curve in Figure 7b.

The model contains 13 parameters describing the mechanical behavior of an aluminum single crystal, such as the three elastic constants $C_{11}$, $C_{12}$, and $C_{44}$; the ten plastic constants: isotropic hardening constants $\tau_{0}$, $\tau_{s}$, $h_{0}$; kinematic hardening constants a, c, and d; cyclic hardening constants $e_{1}$ and $e_{2}$; the reference strain rate ${\dot{\gamma }}_{0}$; the rate sensitivity parameter k.

The three independent elastic constants of single crystal aluminum that characterize the anisotropic elasticity tensor $C_{11}$, $C_{12}$ and $C_{44}$ are obtained from the aluminum elastic properties as a function of elastic modulus E=57 MPa, shear modulus G=25 MPa, which are identified by matching the experimental uniaxial tensile and torsion stress-strain curves in the linear region for this alloy, and Poisson’s ratio μ, which is prescribed as ν=0.3. The reference strain rate ${\dot{\gamma }}_{0}$ is prescribed as 0.001/s^−1^, which is assumed to the rate-independent material. The rate sensitivity parameter k is set to 200, since the pure aluminum is almost strain rate insensitive. The parameter d is set to 0, since creep is ignored during cyclic loading. Using these values, the remaining plastic parameters of crystalline plasticity model, namely, $\tau_{0}$, $\tau_{s}$, $h_{0}$, a, c, $e_{1}$, and $e_{2}$ are calibrated by fitting the stress–strain responses by the experiments under cyclic tension-compression loading, as shown in Figure 7a. The initial critical resolved value $\tau_{0}$ and its saturated value $\tau_{s}$ of shear stress are obtained from the elastic range from the peak points unloading to reverse yielding points in the initial and stable stress–strain hysteresis loop, respectively. For cyclic hardening is less pronounced, the material constant $\tau_{0}$ is close to its saturated value $\tau_{s}$. The parameter $h_{0}$ is the initial hardening rate that describes the hardening rate of materials after they enter plasticity, a is the initial slipping hardening modulus and c is a constant leading to soften, $e_{1}$ and $e_{2}$ are the parameters to describe cyclic hardening rate and saturation rate of the stress-strain hysteresis curve, respectively. Further, the applied material parameters are adjusted repeatedly until the simulated stress–strain stabilized loop and tensile curve are well-matched with the experimental results in Figure 7. It is obvious that the computationally obtained cyclic stress-strain loop is in good agreement with the experimental results, verifying that the parameters used in crystal plasticity model are enough accurate. The finial elastic and plastic property parameters used in the crystal plasticity model are summarized and listed in Table 2.

### 4.3. The Number of Grain effects on the Macroscopic Behavior

**Figure 8:**
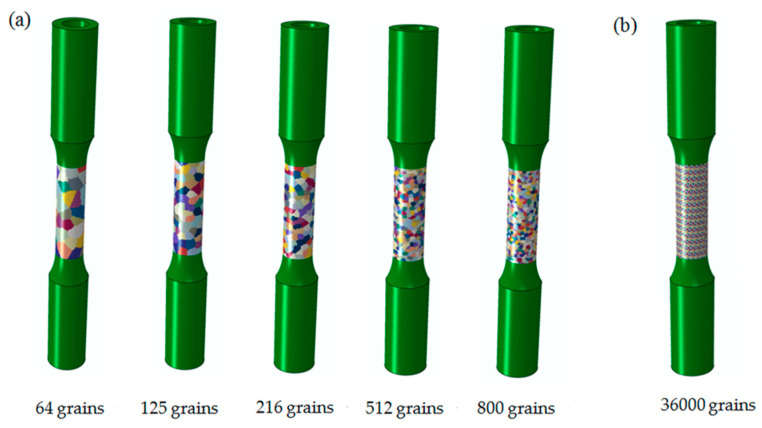
The center gauge region of the GFEM with different grain numbers: (**a**) containing 64, 125, 216, 512, and 800 grains for each grain composed of many elements; (**b**) containing 36,000 grains for each element representing one grain.

Before proceeding further, the number of grains in the center gauge region of the GFEM should be enough to verify the convergence of the predicted results due to random grain orientation, size, and random morphology. However, the computational cost increases heavily with the number of grains, so an optimal number of grains should be discussed for the accurate predictions. For this reason, the center gauge region of the GFEM containing 64, 125, 216, 512, and 800 grains as shown in Figure 8a are studied, respectively. Each grain shown as a single color is a cube containing many elements. Figure 8b displays relatively finer grain size, which is slightly bigger than measured in the center gauge region of the GFEM containing 36,000 grains, each element represents one grain with a given orientation characterized by the color code.

**Figure 9:**
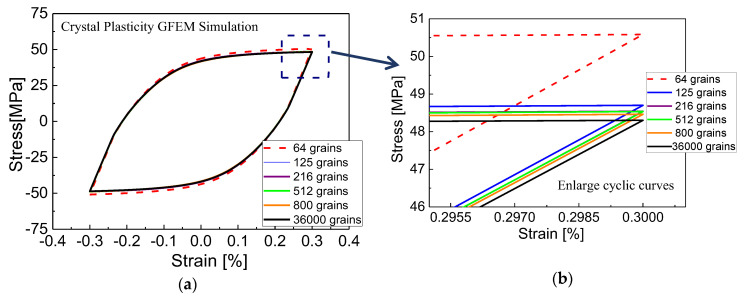
Comparison of steady hysteresis loops under cyclic tension-compression loading simulated by thin-walled tube specimen with different number of grains: (**a**) steady hysteresis loops with crystal plasticity GFEM simulation; (**b**) the enlarge graph of steady hysteresis loops.

The computationally obtained stress–strain cyclic curves for the GFEM with a different number of grains by crystal plasticity under cyclic tension-compression loading are given in Figure 9. It also predicts a slightly higher stress less than 2 MPa with 64 grains than that other grains. It is obvious that computational results of the stress–strain curves almost converge for the center gauge region of the GFEM containing more than 125 grains. From this sensitivity study, it is found that the GFEM with 125 grains are employed to show a converged macroscopic stress–strain response. This insensitivity of numerically computed mechanical behavior to the number of grains is resulted from the randomly arranged orientation and size of grains. The systematically predicted macroscopic stress–strain curves using the models with each grain containing many elements are more closely approximated to that adopting the model with each element representing one grain. Therefore, the thin-walled finite element model in the center gauge region which contains a total of 36,000 elements and 36,000 grains can be adopted in the following computations.

## 5. Results and Discussions

**Figure 10:**
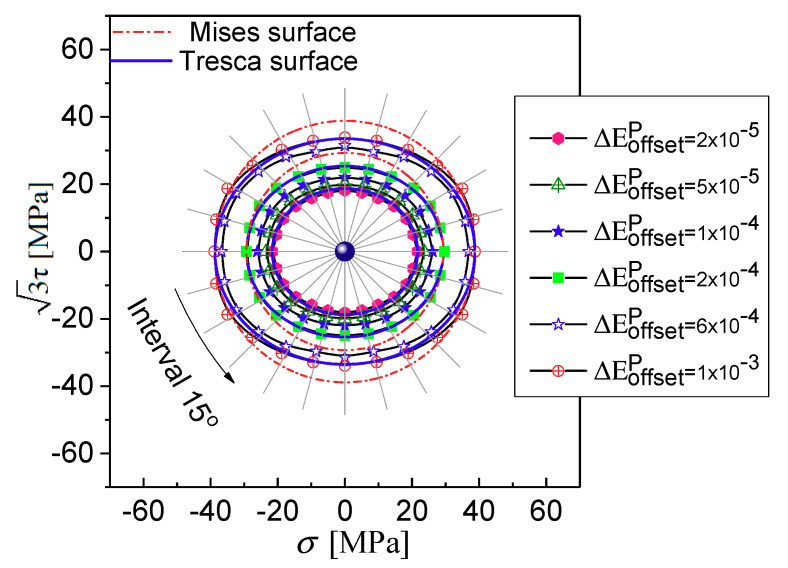
Initial yield surface by the GFEM simulation.

A family of the initial yield surfaces is determined by radial proportional stress-controlled paths with different ratio of shear stress $\sqrt{3}\tau$ to tensile stress σ ranging from 0° to 360° with 15° intervals with the different offset strain by using the GLBM, as shown in Figure 10. Firstly, we observe that the yield surfaces are close to isotropic expansion in the (σ, $\sqrt{3}\tau$) plane with the increasing offset strains whose shape is smooth convex ellipses with two axes of symmetry: the tensile stress axis σ and the shear stress axis $\sqrt{3}\tau$. The shape of the initial yield surfaces is independent of the yield definition choice, but the size of the initial yield surfaces is dependent on the various offset strain values. By comparison, for instance, the tensile and reverse compressive yield stress (38.8, −38.8 MPa) are higher than the shear yield stress and reverse shear (33.87, −33.87 MPa) for a specified offset strain of 1000 με. The initial yield surface of the polycrystal aluminum may be described by the Tresca yield criterion.

With reference to Figure 4, the different reloading paths in the (σ, $\sqrt{3}\tau$) plane among 0° to 180° are adopted to probe the experimental subsequent yield surfaces at the beginning of the unloading points O_A1_ (and O_A2_, the reverse yielding point), O_B_, O_C_, and O_D_ within the elastic domain as the assumed yield surface center corresponding to the four different positions A, B, C, and D, respectively, after 30 symmetry tension-compression cycles at 0.3% strain amplitude under strain control. Furthermore, the experimental results are compared with the predicted ones by the crystal plasticity simulation using the GFEM containing 36,000 elements and 36,000 crystalline grains in the gauge section of a specimen and the RVE containing 27,000 elements and 125 equiaxed grains, and the differences of subsequent yield surfaces predicted between the two finite element models (FEM) with different scales and boundary conditions are discussed.

**Figure 11:**
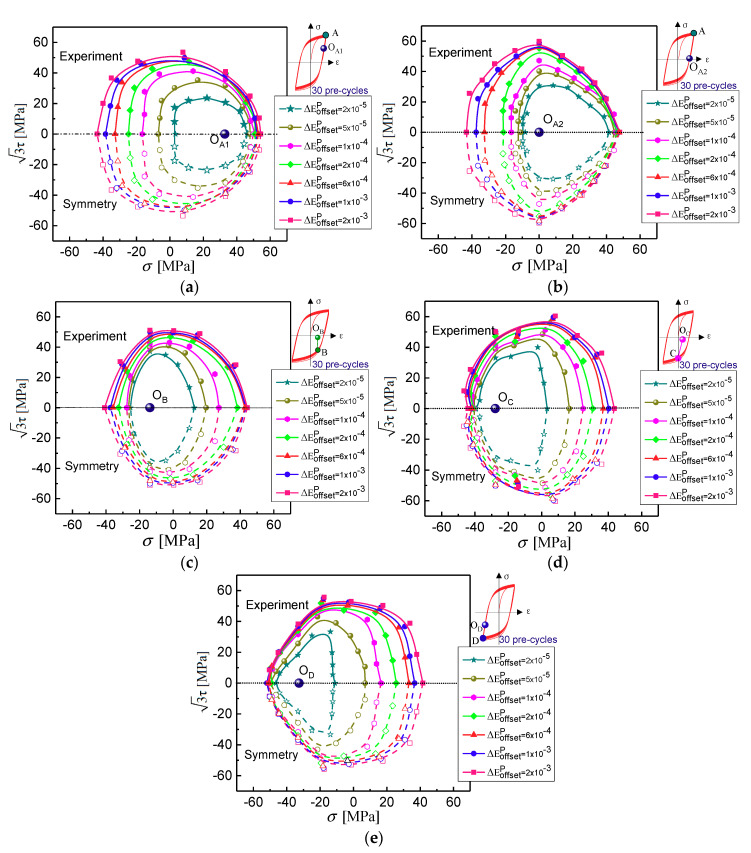
Subsequent yield surface at five different unloading cases from tension to compression transient after 30 tension-compress pre-cycles by experiments. (**a**) unloaded from A to O_A1_; (**b**) unloaded from A to O_A2_; (**c**) unloaded from B to O_B_; (**d**) unloaded from C to O_C_; (**e**) unloaded from D to O_D_.

The experimental subsequent yield surfaces under the above five different unloading cases are plotted in Figure 11, the distinct features of the yield surface for anisotropic yielding can be observed. The subsequent yield surfaces expand rapidly towards the reverse loading direction and shrinks in the direction perpendicular to the pre-loading direction but has little influence in pre-deformation direction. This suggests a rapid hardening of slip under the reverse loading condition and a slow hardening in the preloading direction that dominates deformation under tension (or compression). This is the cause of the higher curvature of yield surface in the pre-loading direction than that in the other directions, and as a result, the subsequent yield surfaces exhibit the “sharp corner” in the pre-deformation direction and flat in its reverse direction. The anisotropic hardening characteristics of the subsequent yield surface strongly depend on the direction of the accumulated plastic strain, which is verified that the “sharp corner” of yield surfaces points to the pre-deformation direction corresponding to tension or compression, seen Figure 11, and their expansion and translation in the stress space are clearly observed. The yield surfaces are approximately symmetric about the stress axis σ located in the pre-loading direction. In Figure 11a,b, the effects of the unloading points O_A1_ and O_A2_ on the evolution of the yield surfaces are displayed. By comparison of the different unloading points, the results of the numerical and experimental study reveal that the yield surface distortion in the pre-loading direction becomes more evident owing to the unloading range increased (A-O_A2_) and the size of yield surfaces becomes shrinkage due to the small reverse plastic deformation at O_A2_ as the smaller offset strain of 20 με and 50 με, the yield surface in stress space is also translated observably.

As mentioned above, the size and shape of the subsequent yield surface depend on the yield definition and, in fact, similar experimental observations were reported in the literature [41,42]. The subsequent yield surfaces will exhibit pronounced kinematic and distorted hardening with a relatively small offset strain. The distorted evolution is characterized by the yield locus with a rounded triangle vertex in the loading direction and perfectly flat opposite to the loading direction. If a relatively large target deviation strain is chosen, for instance, in Figure 11, 200 με, 600 με, and 1000 με, the subsequent yield surfaces exhibit approximately isotropic hardening.

**Figure 12:**
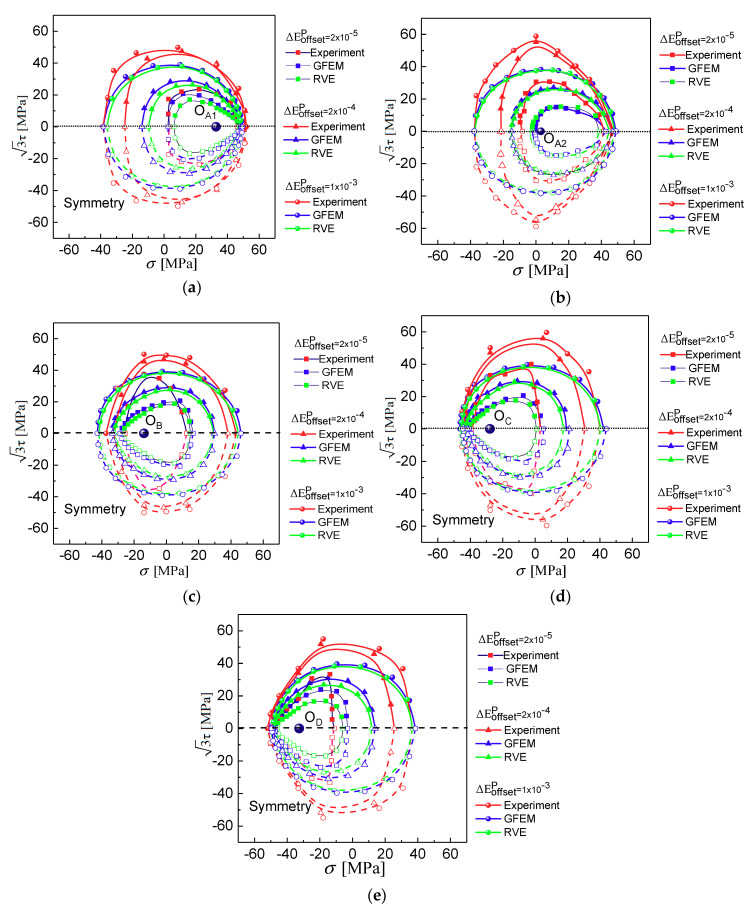
Subsequent yield surface tested and simulated by test and crystal plasticity with the GFEM and RVE after 30 tension–compression pre-cycles. (**a**) unloaded from A to O_A1_; (**b**) unloaded from A to O_A2_; (**c**) unloaded from B to O_B_; (**d**) unloaded from C to O_C_; (**e**) unloaded from D to O_D_.

As shown in Figure 12, the computationally obtained and experimentally measured subsequent yield surfaces are compared, and the trend of subsequent surface predicted is consistent with the experimental observations. It can be seen that the sizes of the yield surfaces have some differences between them, but the distinct features of the yield surfaces for anisotropic yielding can be accurately predicted. It demonstrates that the crystal plasticity model considering the microstructure heterogeneities and slipping mechanism within the crystal can accurately describe the anisotropic evolution of the subsequent yield surfaces caused by pre-loading deformation.

To verify the predictive capabilities of the approach under various levels of the finite element models (FEM) from a micro material point (RVE) to macro specimen (GFEM) scale transition, the first scheme adopted here is the GFEM to simulate a same test specimen with the constrained and loaded conditions corresponding to the axial and torsional deformation history consistent with experiments. In the second scheme, the polycrystalline aggregate RVE model containing 125 grains is adopted to simulated a material point of specimen gauge section, and to reflect the continuity of deformation during loading, the RVE model is applied with the periodic boundary conditions, the macroscopic deformation is controlled by applying displacement and load on the master node of the model, as shown in Figure 6b. The subsequent yield surfaces at five unloading cases with different offset strain are presented with the GFEM and RVE models in Figure 12. As a compromise between accuracy and computational cost, the response of macroscopic deformation is believed to be better approximated when the GFEM is adopted in this study, whereas the RVE model provides a computationally cheap estimate for the polycrystalline response although it expects to be relatively inaccurate. The GFEM systematically predicts the yield surfaces which are slightly larger than the ones by RVE, as it corresponds to the same offset strain due to the development of lattice preferred orientations. As a consequence, there is a nearly indistinguishable difference in the predicted subsequent yield surfaces between the GFEM and the RVE model. The coincidence is attributed to the low sensitivity of finite element model sizes.

**Figure 13:**
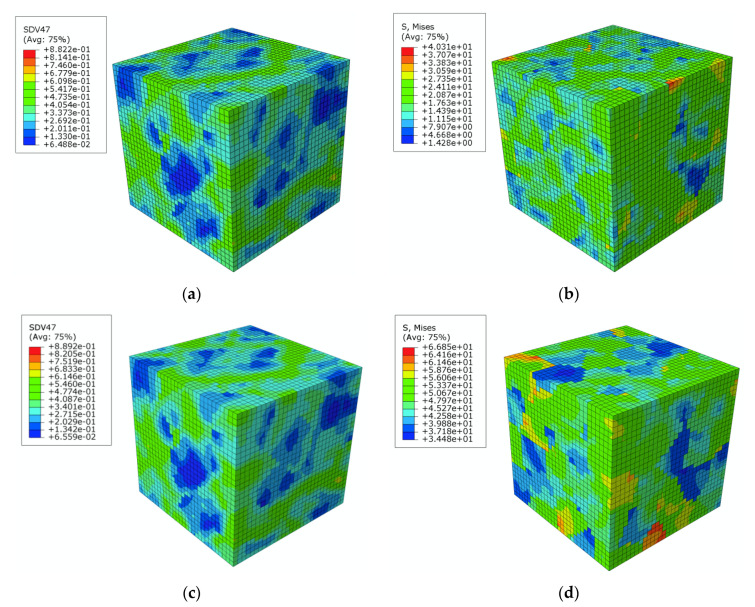
The accumulated equivalent plastic strain and Mises stress contours of a 3D Voronoi polycrystalline aggregate with offset strain of 20 μm and 1000 μm along 180° reloading path at unloading point D-O_D_ after 30 tension-compression cycles: (**a**) equivalent plasticity strain contour with 20 με offset strain; (**b**) Mises stress contour with 20 με offset strain; (**c**) equivalent plasticity strain contour with 1000 με offset strain; (**d**) Mises stress contour with 100 με offset strain.

The statistical analysis about the inhomogeneity of the Representative Volume Element (RVE) with micro-structure randomly generated is extremely important for the physical nature of macroscopic mechanical respond for polycrystalline material undergoing different deformation. In this work, subjected to the deformations of 20 με and 1000 με at the reloading direction of 180°, namely, the lower limiting value and upper limit value of yield definition in this work. The contour distribution of the accumulated equivalent plasticity strain and Mises stress in the RVE by simulation using the crystal plasticity model are displayed in Figure 13, where heterogeneous distribution of the two variables can be clearly observed.

**Figure 14:**
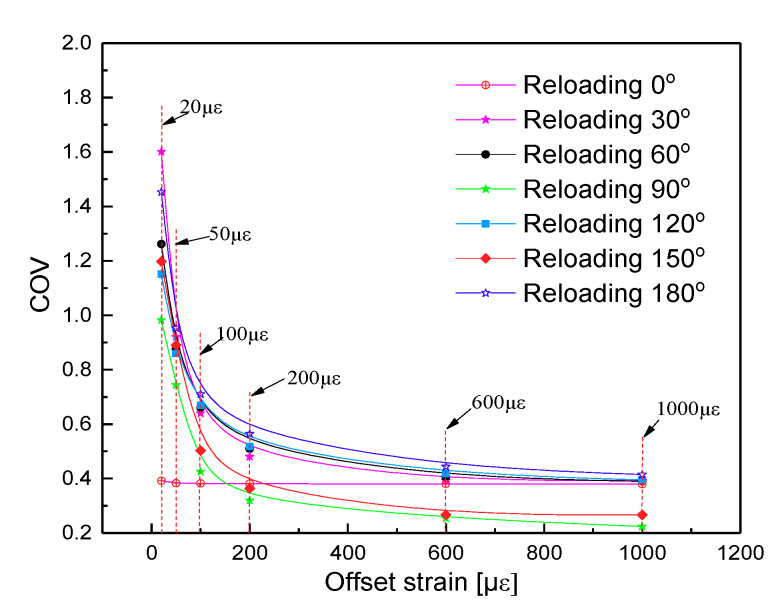
Variation curves of COV (Here, COV was defined as the ratio of the standard deviation S of the accumulated effective plastic strain increment to its average absolute value) for the equivalent plastic strain increment vs offset strain along different reloading paths at unloading point D-O_D_ after 30 tension-compression cycles.

**Figure 15:**
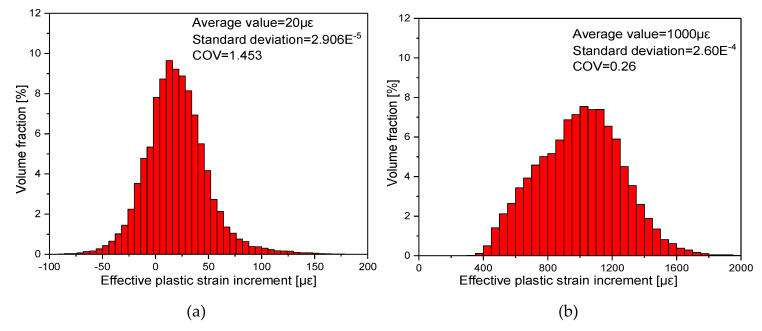
Statistical distribution of micro equivalent plastic strain increment with different offset strain value of 20 με and 1000 με along 180° reloading path at unloading point D-O_D_ after 30 tension-compression cycles. (**a**) the offset strain 20 με; (**b**) the offset strain 1000 με.

To compare the inhomogeneity of equivalent plastic strain increment with different offset strain, a coefficient of variation (COV) was defined as the ratio of the standard deviation S of the accumulated effective plastic strain increment to its average absolute value [10]. Figure 14 illustrates the relation curves between the COV of the equivalent plastic strain increment and offset strain ranging from 20 με to 1000 με along various reloading paths from 0° to 180° with interval 30° at unloading point D-O_D_ after 30 tension-compression cycles. The results show that heterogeneous distributions of strain and stress can be clearly observed in Figure 14 with increasing deformation as accumulated equivalent plasticity strain increments, respectively. For example, the COV of the equivalent plastic strain increment corresponding to the target offset strain of 20 με and 1000 με decreases from 1.453 to 0.26 at the reloading direction of 180° and the statistical-numerical distribution of the equivalent plastic strain increment are shown in Figure 15. These results demonstrate that with a very small offset strain, the representative volume element (RVE) presents very serious heterogeneity leading to anisotropy distortion of the yield surface due to a small amount of elements and grains generating new plastic strain in the RVE. However, if the larger offset strain is chosen, numerous elements and grains occur with new plastic deformation in the RVE, which leads to the plastic deformation of material tending to be homogeneous. By comparison with the same offset strain, as shown in Figure 14, the higher heterogeneity in the preloading direction is revealed than that towards the reverse loading direction. Therefore, no violation of the convexity can be regarded as a requirement for choosing the proper specified offset strain.

## 6. Conclusions

In this work, the crystal plasticity model considering the back-stress associated with the finite element models (FEM) approaches including a micro material point (RVE) and macro specimen (GFEM) at different length scales of polycrystalline aggregates is adopted to validate the predictive capabilities for the subsequent yield surfaces of polycrystal aluminum, and the simulated results are assessed by their comparison with experimental observations. Conclusions are summarized as follows:

1. The shape of the initial yield surfaces is independent of the choice of the yield definition, but the size of the initial yield surfaces is strongly associated with the various offset strain. The initial yield surface of the polycrystal aluminum can be described by the Tresca yield criterion.

2. The directional hardening characteristics of the subsequent yield surfaces for the polycrystal aluminum are strongly associated with the direction of the accumulated plastic strain, which expands rapidly towards the reverse loading direction and shrinks in the direction orthogonal to the preloading direction but has little influence in the pre-deformation direction. Therefore, the subsequent yield surfaces exhibit the “sharp corner” in the pre-deformation direction and flat in the reverse direction.

3. Unlike the initial yield surfaces, the size and shape of the subsequent yield surfaces are obviously sensitive to the offset strain. The subsequent yield surfaces present pronounced kinematic hardening (Bauschinger effect) and distortion with a relatively small offset strain, which leads to a triangle vertex in the direction of loading but flat in the reverse loading direction. If a relatively large offset strain is chosen, the subsequent yield surfaces seem to the isotropic hardening.

4. The yield surface calculated by using the GFEM is believed to be better approximated whereas the RVE model requires a lower computational cost. There is a nearly indistinguishable difference regarding the simulation of subsequent yield surfaces between the two different scale models. We attribute this coincidence to the low sensitivity of finite element model scales.

## Figures and Tables

**Table 1:** Chemical compositions and mechanical properties of polycrystalline aluminum.

Chemical Composition						Mechanical Properties				
Al/%	Cu/%	Mg/%	Si/%	Mn/%	Zn/%	E/GPa	G/GPa	$\sigma_{0.2}/\mathrm{MPa}$	$\sigma_{u}/\mathrm{MPa}$	$\varepsilon_{f}$
99.89	0.02	0.03	0.03	0.02	0.01	57	25	20	81	24%

**Table 2:** The material constants of single crystal aluminum.

$C_{11}$	$C_{12}$	$C_{44}$	$\tau_{0}$	$\tau_{s}$	$h_{0}$	a	c	$e_{1}$	$e_{2}$	d	${\dot{\gamma }}_{0}$	k
/GPa	/GPa	/GPa	/MPa	/MPa	/MPa	/GPa	/GPa				/s^−1^	
75.64	36.86	25.4	8.42	9.2	65	22.5	2.5	0.1	1.0	0	0.001	200

## References

[1] Ohashi Y., Kawashima K., Yokochi T. (1975). Anisotropy due to plastic deformation of initially isotropic mild steel and its analytical formulation. J. Mech. Phys. Solids.

[2] Kuwabara T., Yoshida K., Narihara K., Takahashi S. (2005). Anisotropic plastic deformation of extruded aluminum tube under axial forces and internal pressure. Int. J. Plast..

[3] Wu H.C., Yeh W.C. (1991). On the experimental determination of yield surfaces and some results of annealed 304 stainless steel. Int. J. Plast..

[4] Phillips A., Lu W.Y. (1984). An experimental investigation of yield surfaces and loading surfaces of pure aluminum with stress-controlled and strain-controlled paths of loading. J. Eng. Mater. Technol..

[5] Khan A.S., Kazmi R., Pandey A., Stoughton T.B. (2009). Evolution of subsequent yield surfaces and elastic constants with finite plastic deformation. Part-I: A very low work hardening aluminum alloy (Al6061-T6511). Int. J. Plast..

[6] Khan A.S., Pandey A., Stoughton T. (2010). Evolution of subsequent yield surfaces and elastic constants with finite plastic deformation. Part II: A very high work hardening aluminum alloy (annealed 1100 Al). Int. J. Plast..

[7] Khan A.S., Pandey A., Stoughton T. (2010). Evolution of subsequent yield surfaces and elastic constants with finite plastic deformation. Part III: Yield surface in tension–tension stress space (Al 6061–T 6511 and annealed 1100 Al). Int. J. Plast..

[8] Sung S.J., Liu L.W., Hong H.K., Wu H.C. (2011). Evolution of yield surface in the 2D and 3D stress spaces. Int. J. Solids Struct..

[9] Zhang K., Badreddine H., Saanouni K. (2018). Thermomechanical modeling of distortional hardening fully coupled with ductile damage under non-proportional loading paths. Int. J. Solids Struct..

[10] Zhang K.S., Shi Y.K., Xu L.B. (2011). Anisotropy of yielding/hardening and microinhomogeneity of deforming/rotating for a polycrystalline metal under cyclic tension-compression. Acta Metall. Sin..

[11] Kowalewski Z.L., Sliwowski M. (1997). Effect of cyclic loading on the yield surface evolution of 18G2A low-alloy steel. Int. J. Mech. Sci..

[12] Rokhgireh H., Nayebi A., Chaboche J.L. (2017). Application of a new distortional yield surface model in cyclic uniaxial and multiaxial loading. Int. J. Solids Struct..

[13] Parma S., Pleek J., Marek R., Hruby Z., Feigenbaum H.P., Dafalias Y.F. (2018). Calibration of a simple directional distortional hardening model for metal plasticity. Int. J. Solids Struct..

[14] Stout M.G., Martin P.L., Helling D.E., Ganova G.R. (1985). Multiaxial yield behavior of 1100 aluminum following various magnitudes of prestrain. Int. J. Plast..

[15] Kan A.S., Wang X. (1993). An experimental study on subsequent yield surface after finite shear pre-straining. Int. J. Plast..

[16] Ishikawa H., Sasaki K. (1988). Yield surfaces of SUS304 under cyclic loading. J. Eng. Techonol..

[17] Ishikawa H. (1997). Subsequent yield surface probed from its current center. Int. J. Plast..

[18] Baltov A., Sawczuk A. (1965). A rule of anisotropic hardening. Acta Mech..

[19] Dafalias Y.F., Schick D., Tsakmakis C. (2002). A simple model for describing yield surface evolution during plastic flow. Appl. Comput. Mech..

[20] Chen J.Y., Zhang K.S., Kuang Z., Hu G.J., Song Q., Chang Y.J. (2019). The anisotropic distortional yield surface constitutive model based on the chaboche cyclic plastic model. Materials.

[21] Francois M. (2001). A plasticity model with yield surface distortion for non proportional loading. Int. J. Plast..

[22] Barlat F., Gracio J.J., Lee M.G., Rauch E.F., Vincze G. (2011). An alternative to kinematic hardening in classical plasticity. Int. J. Plast..

[23] Feigenbaum H.P., Dafalias Y.F. (2007). Directional distortional hardening in metal plasticity within thermodynamics. Int. J. Solids Struct..

[24] Lian J.S., Chen J.W. (1991). Isotropic polycrystal yield surfaces of b.c.c. and f.c.c. metals: Crystallographic and continuum mechanics approaches. Acta Metall. Mater..

[25] Daehli L.E.B., Hopperstad O.S., Benallal A. (2019). Effective behaviour of porous ductile solids with a non-quadratic isotropic matrix yield surface. J. Mech. Phys. Solids.

[26] Hu G.J., Huang S.H., Lu D.M., Zhong X.C., Li Z.H., Wolfgang B., Zhang K.S. (2015). Subsequent yielding of polycrystalline aluminum after cyclic tension-compression analyzed by experiments and simulations. Int. J. Solids Struct..

[27] Zhang K.S., Huang S.H., Liu G.L., Lu D.M. (2017). Measuring subsequent yield surface of pure copper by crystal plasticity simulation. Chin. J. Theor. Appl. Mech..

[28] Lee E.H. (1969). Discussion: “Elastic-plastic deformation at finite strains”. J. Appl. Mech..

[29] Hill R., Rice J.R. (1972). Constitutive analysis of elastic–plastic crystals at arbitrary strain. J. Mech. Phys. Solids.

[30] Asaro R.J., Rice J.R. (1977). Strain localization in ductile single crystals. J. Mech. Phys. Solids.

[31] Needleman A., Asaro R.J., Lemonds J., Peirce D. (1985). Finite element analysis of crystalline solids. Comput. Meth. Appl. Mech. Eng..

[32] Zhang K.S., Shi Y.K., Ju J.W. (2013). Grain-level statistical plasticity analysis on strain cycle fatigue of a FCC metal. Mech. Mater..

[33] Peirce D., Asaro R.J., Needleman A. (1983). Material rate dependence and localized deformation in crystalline solids. Acta Metall..

[34] Rice J.R. (1971). Inelastic constitutive relations for solids: An internal variables theory and its application to metal plasticity. J. Mech. Phys. Solids.

[35] Hutchinson J.W. (1976). Bounds and self-consistent estimates for creep of polycrystalline materials. Proc. R. Soc. A Math. Phys..

[36] Hutchinson J.W. (1970). Elastic-plastic behaviour of polycrystalline metals and composites. Proc. R. Soc. A Math. Phys..

[37] Chang Y.W., Asaro R.J. (1981). An experimental study of shear localization in aluminum-copper single crystals. Acta Metall..

[38] Walker K.P. (1981). Research and Development Program for Non-linear Structural Modeling with Advanced Time-temperature Dependent Constitutive Relationships.

[39] Chaboche J.L. (1991). On some modifications of kinematic hardening to improve the description of ratchetting effects. Int. J. Plast..

[40] Segurado J., Llorca J. (2013). Simulation of the deformation of polycrystalline nanostructured Ti by computational homogenization. Comput. Mater. Sci..

[41] Phillips A., Juh-Ling T. (1972). The effect of loading path on the yield surface at elevated temperatures. Int. J. Solids Struct..

[42] Moon H. (1976). An experimental study of the outer yield surface for annealed polycrystalline aluminium. Acta Mech..

